# Orally administered extracellular vesicles from *Salmonella*-infected macrophages confer protective immunity *in vivo*

**DOI:** 10.3389/fimmu.2025.1628756

**Published:** 2025-08-15

**Authors:** Saloni Bhimani, Jorge J. Canas, Samantha M. Enslow, Ryan Mulcare, Mariola J. Ferraro

**Affiliations:** ^1^ Department of Microbiology and Cell Science, Institute of Food and Agricultural Sciences, University of Florida, Gainesville, FL, United States; ^2^ The Department of Molecular Genetics and Microbiology, College of Medicine, University of Florida, Gainesville, FL, United States

**Keywords:** exosomes, extracellular vesicle (EV), *Salmonella* Typhimurium, macrophage, oral vaccination campaigns, oral vaccination

## Abstract

*Salmonella* is a leading cause of foodborne illness in the United States and worldwide. This enteric pathogen deploys various mechanisms to evade the intestinal mucosal barrier to enhance its survival and further infect systemic tissues. Commercially available vaccines against *Salmonella* are currently restricted to the serovar Typhi, while none are currently approved for non-typhoidal *Salmonella* (NTS) serovars, which are becoming increasingly resistant to antibiotics. Due to the lack of effective vaccines against NTS infections, novel oral vaccination strategies have garnered significant interest, owing to their protective abilities at the susceptible sites of infection. We previously reported that mice immunized intranasally with small extracellular vesicles (sEVs) derived from *Salmonella-*infected macrophages protect mice against lethal *Salmonella* challenge. In the present study, we used an oral route of administration of sEVs to determine their protective abilities *in vivo.* Remarkably, orally administered sEVs from *Salmonella-*infected macrophages conferred significant host protection, marked by improved survival post-challenge and reduction in tissue bacterial burdens. Additionally, immunized mice exhibited robust serological responses, including elevated levels of both whole-*Salmonella* and OmpA-specific IgG antibodies. Collectively, these findings show the potential of orally delivered sEVs as a promising, cell-free vaccine platform for protection against salmonellosis.

## 1 Introduction

The global burden of *Salmonella* expands beyond the commonly known typhoid fever caused by *Salmonella* Typhi, the leading cause of bloodstream infections in Asia. Salmonellosis caused by non-typhoidal serovars of *Salmonella* (NTS), including *Salmonella enterica* serovar Typhimurium, often leads to self-limiting gastroenteritis in individuals with healthy immune systems. However, in immunocompromised individuals, NTS can cause bacteremia resulting in significant morbidity and mortality (1). In the United States alone, NTS is responsible for approximately 1.4 million infections per year and is one of the leading causes of foodborne illness, as reported by the CDC (2). *Salmonella* utilizes numerous virulence factors at different stages of infection. The first step of infection involves invasion of M cells present at mucosal sites, for which the Gram-negative bacterium utilizes *Salmonella* pathogenicity island 1 (SPI-1) encoded effector proteins, while SPI-2 effectors predominantly allow for establishment of an intracellular niche within the *Salmonella* containing vacuole (SCV). To prolong its survival within the host, *Salmonella* has adapted to the epithelial cell shedding process which allows this pathogen to disseminate into the intestinal lumen, in turn infecting a larger number of resident and non-resident cells (3).

Although licensed vaccines such as Ty21a and Vi-PS are available for *S.* Typhi, no approved vaccine currently exists for human use against NTS. In settings with limited resources, NTS infections often go undiagnosed in immunocompromised individuals. Furthermore, the use of antibiotics such as fluoroquinolones has led to an increase in antimicrobial resistance, thus prioritizing the development of a safe and effective vaccine (4). Mouse models using *S.* Typhimurium effectively resemble features of systemic typhoid-like disease, enabling detailed investigations of host-pathogen interactions (5). The innate immune system represents the first line of defense against *Salmonella*, orchestrating in the primary stages of infection clearance. However, *S.* Typhi virulence can circumvent these antimicrobial responses to persist and colonize host intestinal and systemic sites. While robust innate immune capabilities are activated during *Salmonella* infection, durable long-term immunological protection requires mounting of effective adaptive immune responses (6).

Recently, extracellular vesicles (EVs) have emerged as promising acellular vaccine platforms. EVs are lipid bilayer-enclosed nano- and micro-particles secreted by eukaryotic cells through various biogenesis pathways (7). EVs play an important role in intercellular communication, across both short and long distances and can carry a wide range of bioactive molecules, including proteins, lipids, and nucleic acids (8). EVs are typically categorized into: small EVs (sEVs) ranging from 30–150 nm in size, medium-sized EVs that are around 200–800 nm in diameter, and large EVs that have a diameter greater than 1000 nm and mostly include apoptotic bodies, large oncosomes or exopheres (7). Their capacity to transport antigenic cargo and immunomodulatory molecules positions EVs as attractive candidates for immunotherapeutic applications, particularly in the context of infection (9, 10).

Previous studies conducted by our laboratory have identified *Salmonella-*encoded antigens within sEVs isolated from RAW264.7 macrophages at 24- and 48- hours post-infection (hpi) via mass spectrometry (11). Mice intranasally (IN) receiving sEVs from *Salmonella-*infected macrophages have demonstrated prolonged survival upon *S.* Typhimurium challenge in comparison to the control group (12). In this study, we specifically demonstrate that mice orally immunized with sEVs from *Salmonella-*infected macrophages had better survival upon *S.* Typhimurium challenge. The immunized mice displayed decreased weight loss, improved body scores, and reduced bacterial loads in their tissues. Additionally, the protective efficacy of sEVs was also demonstrated by an elevated production of antigen-specific IgG, providing a platform for evaluating innovative delivery methods of EV-based vaccines.

## 2 Materials and methods

### 2.1 Cell and bacterial culture

RAW264.7 (ATCC TIB-71) murine macrophages were grown and maintained in complete DMEM, supplemented with 10% fetal bovine serum (FBS) and 1% penicillin and streptomycin, at 37°C and 5% CO_2._ *Salmonella* Typhimurium strains UK-1 (χ3761) and Δ*aroA* (χ9099) were grown in lysogeny broth (LB) Miller with constant shaking at 250 rpm at 37°C overnight (14–16 hours). The OD_600_ of the overnight culture was measured using a spectrophotometer and the culture was diluted to an OD600 of 0.05 in 20mL LB miller and grown up to an OD_600_ of 0.5 to use for experiments. Based on a previously established growth curve, the reported OD corresponds to approximately 3.3 × 10^8^ CFU/mL for UK-1 and 6.5 × 10^8^ CFU/mL for Δ*aroA Salmonella*.

### 2.2 Cell infection and EV isolation

RAW264.7 macrophages were grown up to confluency in complete DMEM medium. Prior to infection, cells were washed with 1X phosphate buffered saline (PBS) to remove traces of FBS and antibiotics and replaced with DMEM containing 1% exosome-depleted FBS (exo-free DMEM). Cells were infected with *S.* Typhimurium UK-1 at a multiplicity of infection (MOI) of 5 for 2 hours. Media on cells was then replaced with exo-free DMEM containing 100 μg/mL gentamicin for 1 hour to remove any extracellular *Salmonella.* After one hour, the media was changed again and replaced with exo-free DMEM containing 25 μg/mL gentamicin and cells were allowed to incubate till the cell supernatant was collected for EV isolation 24 hours post infection, and the cell supernatant was replenished for collection 48 hours post infection.

Collected supernatants underwent sequential centrifugation at 500 × g and 4,000 × g for 10 minutes each, followed by 16,000 × g for 30 minutes to remove cell debris. The collected media was filtered through a 0.2 μm filter and ran at 100,000 x g for 3 hours using ultracentrifuge fitted with an SW32Ti rotor (Beckman Coulter Optima XPN-90). After the first run, the media was decanted and the EVs were resuspended in 400uL PBS with 1% protease inhibitor, and the rest of the ultracentrifuge tubes were balanced using sterile 1X PBS. The ultracentrifugation step was repeated and the EVs were finally resuspended in 1mL of PBS with 1% protease inhibitor. The protein concentration was determined using the Pierce BCA kit (Thermo Fisher Scientific). The particle concentration and size were determined using a ZetaView QUATT particle tracking analyzer (Particle Matrix). Three replicates of EVs (10 μg protein/each) from uninfected and infected macrophages were stained with MACSplex EV kit (Miltenyi Biotec Catalog no. 130122211) using manufacturer’s instructions to identify and compare tetraspanin markers. The EVs were analyzed using a flow cytometer (Beckman Coulter cytoFLEX) and FlowJo software.

### 2.3 Western blotting

Western blotting was carried out using 20 μg EVs per sample. Sample buffer containing beta-mercaptoethanol (BME), Deionized (DI) water, and 4 x XT-sample buffer was made and 25 μL sample was loaded per well in a 4-12% bis-tris gel (BioRad). The gel was run at 75V for 15 minutes, after which the voltage was increased to 200V, and the gel was run for another 45 minutes. The gel was then transferred to a blot and stained with primary antibody [1:800 dilution for anti-CD63 (System Biosciences), anti-CD9 (System Biosciences), anti-Alix (System Biosciences), and anti-OmpA (Vector Laboratories)] with overnight shaking at 4°C. The blot was then washed three times with PBST (PBS with 0.1% Tween-20) for 3 minutes each, after which it was stained with an HRP-conjugated goat anti-rabbit secondary antibody (1:500 dilution) for 1–2 hours with constant shaking. The blot was washed with PBST again and enhanced chemiluminescence (ECL) substrate was added for 5 minutes before imaging the blot using iBright Imaging System (Invitrogen).

### 2.4 Mouse experiments

Seven-week-old female BALB/c mice (Jackson Laboratory) were orally dosed with 40 μg sEVs from *Salmonella-*infected macrophages at week 0, 2, and 4. As a negative and positive control, mice were also immunized orally with sterile 1X PBS and *S.* Typhimurium Δ*aroA* (4.5 x 10^6^ CFUs) respectively. Prior to the administration of immunizations, mice were orally dosed with 50 μL of 0.3M sodium bicarbonate using a 22-gauge oral gavage needle to neutralize their stomach acid for standardized *Salmonella* infection. After waiting 10 minutes, another 22-gauge gavage needle was used to administer the appropriate dose resuspended in 100 μL of 1X PBS. For the survival study, mice were orally challenged with a lethal dose (4.5 x 10^6^ CFUs) of *S.* Typhimurium UK-1 following the abovementioned dosing protocol, 7 weeks after their first sEV dose, and their weights and body scores were assessed daily for 30 days or until endpoint. Mice were euthanized in a chamber using CO_2_ at a 30-70% displacement rate, followed by a cervical dislocation as a secondary confirmation of death. For serological analysis, blood was collected via the saphenous vein at weeks 1, 3, 5, and 7. After centrifugation at 10,000 × g for 10 minutes, serum was collected and stored at –20°C until ELISA analysis.

### 2.5 Bacterial burden

To assess the bacterial burden, mice immunized with sEVs or PBS control were challenged with 4.5x10^6^ CFUs of *S.* Typhimurium given orally, 7 weeks after their first EV dose. 4 days post challenge with *Salmonella*, mice were euthanized, and their spleen and liver were collected. The organs were weighed and resuspended in 0.1% Triton-X in PBS and lysed using TissueLyser LT (Qiagen). Serial dilutions were made in PBS and 100 μL volume was plated on LB agar plates. The agar plates were incubated at 37°C overnight after which colonies were counted and CFU per gram of each organ was determined.

### 2.6 Protein purification and mass spectrometry

OmpA gene from *S.* Typhimurium UK-1 was amplified using polymerase chain reaction (PCR), using the forward primer TAAGCAGGATCCAATGAAACTTAAGTTAGTGGCAGTG and reverse primer TAAGCAAAGCTTTTAGAACTGGTAGTTCAGACCAAC. The amplified fragment was ligated into a pET28a plasmid which was digested using EcoRI and HindIII restriction enzymes. The ligated vector was transformed into *E. coli* DH5α cells after which the plasmid was purified and re-transformed into *E. coli* BL21(DE3) cells for protein purification. The 6X His-tagged OmpA was purified using Ni-NTA resin, for which a bacterial culture containing transformed BL21(DE3) cells was grown up to an OD600 of 0.6 which constant shaking at 200 rpm at 28°C. The protein was induced by adding 0.5 mM IPTG to the culture and leaving it overnight in a shaker incubator at 200 rpm at 19°C. After overnight induction the bacterial cells were lysed by sonication and the cell pellet was resuspended and left at room temperature for 15 minutes in 6M urea after centrifugation at 4200 rpm for 10 minutes. The suspension was centrifuged again at 4200 rpm for 20 minutes after which the supernatant was saved for purification of OmpA. Ni-NTA column affinity chromatography was conducted following manufacturer’s instructions (Thermo Fisher Scientific Cat. No. 88228). The eluted sample was run on an SDS-PAGE and was stained with GelCode Blue stain for 2 hours (Thermo Fisher Scientific) and destained with DI water overnight. The resulting OmpA band was excised from the gel, subjected to in-gel trypsin digestion according to standard protocols (11), and analyzed using a timsTOF flex mass spectrometer (Bruker). Peptides were identified via database searching using Mascot (version 2.7.0) against the *Salmonella* Typhimurium database with trypsin as the specified enzyme, a precursor ion tolerance of 20 ppm, and fragment ion tolerance of 0.50 Da. Carbamidomethylation of cysteine was set as a fixed modification, and variable modifications included methionine oxidation, N-terminal acetylation, pyro-Glu formation from glutamine, and deamidation of asparagine and glutamine. Protein identifications were validated using Scaffold (version 5.2.2, Proteome Software Inc.), with peptide and protein thresholds set at 95% confidence, and requiring a minimum of two unique peptides. Identified proteins were grouped by parsimony and confirmed using the Protein Prophet algorithm.

### 2.7 ELISAs

Nunc 96-well plates were coated with 2 μg LPS-detoxified *Salmonella* per well and left at room temperature overnight. On day two, excess antigen was removed from the wells by blotting the plate on paper towels. Blocking buffer (PBS with 1% BSA) was added to the wells and the plate was blocked for 2 hours at 37°C. The plate was washed three times with PBST followed by two washes with PBS. The plate was blotted dry after each wash. Serum dilutions were prepared in ELISA buffer (PBS with 0.5% BSA and 0.05% Tween20) and added to the wells containing *Salmonella* antigen. The plate was refrigerated overnight. On day three, plates were washed as previously described. HRP-conjugated goat anti-mouse IgG (Southern Biotech, Cat. No. 1030-05) diluted 1:2,000 in ELISA buffer was added (50 μL/well), and plates were incubated for 90 minutes at 37°C. After washing, 50 μL ABTS (2,2’-azino-bis (3-ethylbenzothiazoline-6-sulfonic acid)) substrate was added per well and developed for 30 minutes at room temperature. Absorbance was read at 415 nm using a Cytation 3 plate reader (BioTek).

### 2.8 Statistical analysis and figure design

GraphPad Prism 10.3.1 was used for figure rendering, and statistical results are denoted with ns for not significant * for p-value at <0.05, ** at <0.01, *** and **** at p<0.0001 in each graph. For comparing two groups with equal variances, students t-test was applied. For comparing three or more groups, one-way and two-way ANOVA with *post hoc* Tukey’s multiple comparison tests were performed. Schematic diagrams representing study design were created using Biorender.

## 3 Results

### 3.1 sEVs from *Salmonella-*infected macrophages express markers required for antigen presentation

**Figure 1:**
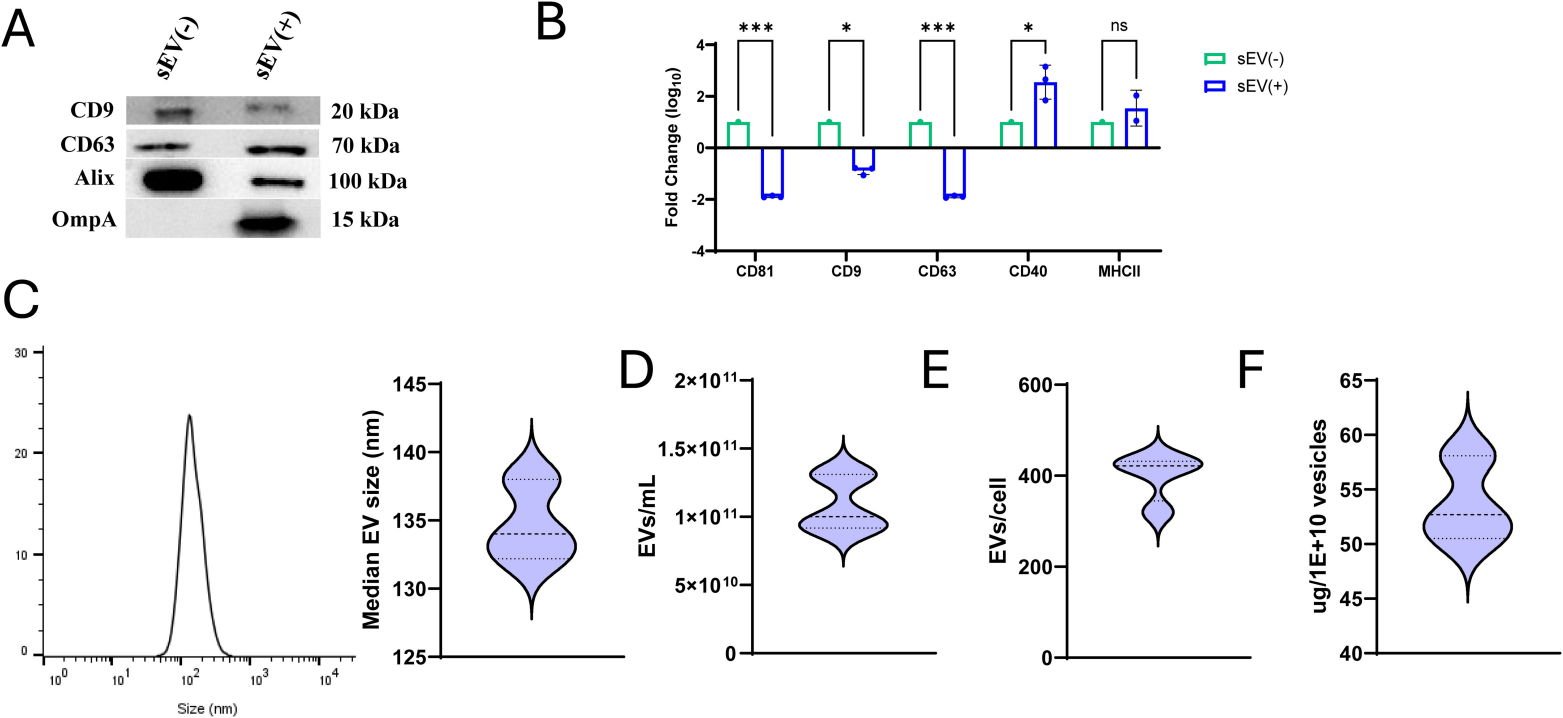
Characterization of small extracellular vesicles (sEVs) derived from *Salmonella*-infected RAW 264.7 macrophages. **(A)** Expression of tetraspanin markers CD63 and CD9, cytosolic marker Alix, and *Salmonella* antigen OmpA in sEVs isolated from uninfected RAW 264.7 macrophages compared to sEVs isolated from macrophages infected with *Salmonella* for 24 and 48 hours (MOI: 5:1). **(B)** Fold change in expression of tetraspanin markers (CD81, CD9, and CD63), CD40 and MHCII between sEVs from uninfected [sEV(-)] and infected [sEV(+)] macrophages quantified using MACSplex EV kit (n=3 independent experiments). Two-way ANOVA with *post hoc* Tukey’s multiple test comparison was used for analysis. Results are denoted with * for p-value at <0.05, *** at <0.001 and ns, not significant. **(C)** Histogram showing sEV size (left), data derived from ZetaView nanoparticle tracking analysis (NTA) and analyzed using FlowJo. Median size of sEVs from triplicate samples (right). **(D)** Quantification of sEVs per mL of culture media (n=5). **(E)** Number of sEVs produced per cell (n=4). **(F)** Protein content (μg) per 1 × 10^10^ vesicles (n=3).

In the present study, we evaluated the immunogenicity and protective efficacy of sEVs administered via the oral route. sEVs were isolated from *S.* Typhimurium-infected macrophages and characterized by standard methods. Western blotting confirmed the presence of canonical sEV tetraspanins CD63 and CD9, and Alix, a cytoplasmic marker. Furthermore, the presence of *Salmonella* antigen OmpA in sEVs was also confirmed (**Figure 1A**). Comparative analysis using the MACSplex EV kit revealed a significant reduction in the tetraspanins CD63, CD81 and CD9 in sEVs derived from *Salmonella*-infected macrophages (obtained at 24- and 48- hours post-infection) relative to uninfected controls. Notably, CD40 expression was upregulated in sEVs from infected cells, while MHCII showed a slight albeit insignificant increase (**Figure 1B**). Nanoparticle tracking analysis (NTA) showed consistent vesicle size distributions and yields across independent isolations (**Figures 1C–F**). The protein-to-particle ratio was determined using a BCA protein assay to calculate the sEV production per cell, which remained reproducible, confirming the reliability of our preparation protocol.

### 3.2 sEV vaccination protects mice against lethal *S.* Typhimurium challenge

**Figure 2:**
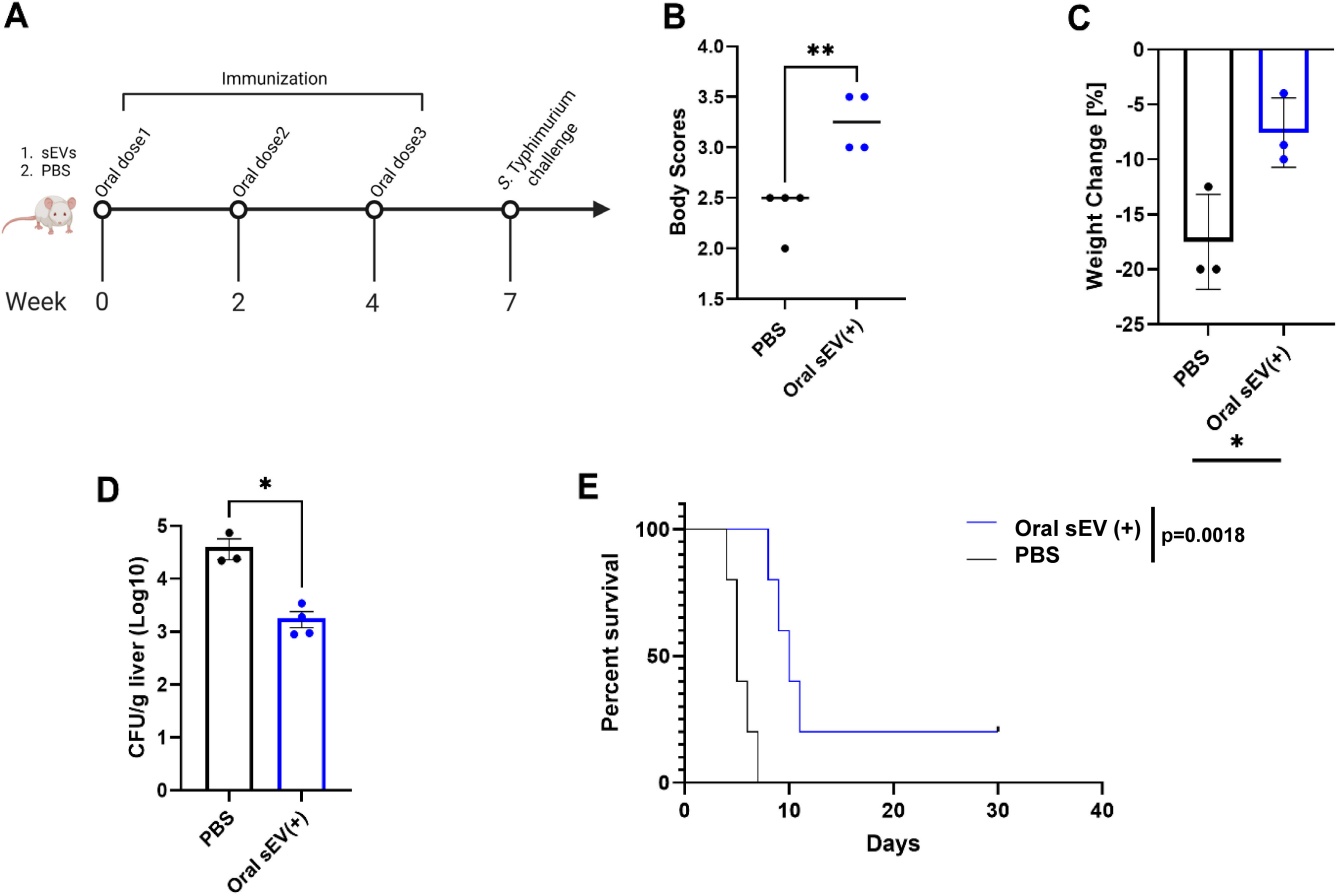
Preventive efficacy of orally administered sEVs from *Salmonella*-infected macrophages against salmonellosis. BALB/c mice were orally immunized with three doses of sEVs (40 µg per dose), or PBS at two-week intervals. Three weeks after the final dose, mice were challenged orally with virulent *S. Typhimurium*. **(A)** Immunization and challenge timeline. **(B)** Clinical disease scores based on body condition assessments on day 4 post-challenge (n=4). Unpaired parametric t-test used for analysis. **(C)** Percent body weight change on day 4 post-challenge (n=3). Unpaired parametric t-test used for analysis. **(D)** Bacterial burden in the liver (CFU/g) four days post-challenge. Unpaired parametric t-test used for analysis. **(E)** Kaplan–Meier survival curves over 30 days post-challenge (n=5). Results are denoted with * for p-value at <0.05, ** at <0.01.

To evaluate the protective efficacy of the oral dose of sEVs derived from *Salmonella*-infected macrophages, 7-week-old BALB/c mice were immunized with sEVs (40 µg per dose) or phosphate-buffered saline (PBS) as a negative control. Our previous studies have already established a protective response to immunization with a positive control – an attenuated *Salmonella* strain – *S.* Typhimurium Δ*aroA* (χ9099), delivered orally (12). Mice received three doses at two-week intervals. Three weeks following the final booster, mice from both groups were challenged with a lethal dose of *S.* Typhimurium UK-1 (χ3761) delivered using oral gavage (**Figure 2A**). Clinical symptoms were monitored daily through body condition scoring and weight measurement. Upon challenge with virulent *S.* Typhimurium, sEV-immunized mice showed improved body condition scores (**Figure 2B**), reduced weight loss (**Figure 2C**), and significantly lower bacterial burdens in the liver at day 4 post-infection (**Figure 2D**), Moreover, survival analysis revealed a significantly higher survival rate in the sEV-treated group compared to controls over 30 days post-infection with *Salmonella* (**Figure 2E**).

### 3.3 Immunization with sEVs leads to production of antigen-specific IgG

**Figure 3:**
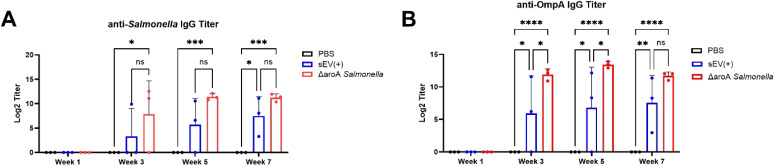
Serum IgG titers measured over time post-immunization. BALB/c mice were orally immunized with three doses of sEVs (40 µg per dose), *Salmonella* Δ*aroA* mutant strain, or PBS at two-week intervals. Blood was collected each week after immunization, at weeks 1, 3, 5, and 7, prior to lethal challenge with *S.* Typhimurium. **(A)** Serum anti-*Salmonella* IgG levels measured at multiple time points during immunization (n=3). Log2 titers were defined as the reciprocal of the dilution giving absorbance 0.1U above absorbance. **(B)** Serum anti-OmpA IgG levels measured at multiple time points during immunization (n=3). Log2 titers were defined as the reciprocal of the dilution giving absorbance 0.5U above absorbance. Two-way ANOVA with *post hoc* Tukey’s multiple comparison tests were used for analysis. Results are denoted with * for p-value at <0.05, ** at <0.01, *** and **** at p<0.0001. ns, not significant.

Mice that received three oral doses of sEVs exhibited elevated serum IgG levels against LPS-detoxified *Salmonella* lysate compared to PBS controls. Additionally, sEV immunized mice showed comparable IgG production, beginning three weeks all the way up to seven weeks after immunization, to the positive control *Salmonella* Δ*aroA*, affirming the efficacy of our sEVs over a long period of time (**Figure 3A**). Due to various *Salmonella* antigens being encapsulated and enriched in sEVs from *Salmonella-*infected macrophages, we predicted that immunized mice could produce serum IgG to a known immunodominant protein OmpA. To demonstrate this, recombinant *S.* Typhimurium OmpA was purified using affinity chromatography and the sequence was confirmed via mass spectrometry (**Supplementary Figure S1**). Mice immunized with sEVs produced significantly higher levels of OmpA-specific serum IgG as compared to the control group over a period of seven weeks (**Figure 3B**). These findings demonstrate that sEVs derived from *S.* Typhimurium-infected macrophages, when delivered orally, can confer significant protection against lethal *Salmonella* challenge. Results from our current study expand on previous work utilizing intranasal delivery and highlight the potential of sEVs as an orally deliverable vaccine platform.

## 4 Discussion

While several delivery routes of therapeutics have been explored, the reduced invasiveness and improved feasibility of administration through the oral route have been established over the years, highlighting their importance for drug delivery (13). Oral vaccines are of significant interest due to their ability to induce both systemic and mucosal immune response. However, effective mucosal immunization faces several challenges due to the degradative environment and tolerogenic immune responses of the oral mucosa, stomach, and small intestine (14). Ty21a – an orally delivered live attenuated *S.* Typhi vaccine – is formulated with an enteric coating to bypass digestive enzymes within the stomach (15). Birds immunized with a mannose chitosan nanoparticle-based vaccine have demonstrated induction of mucosal immunity against *Salmonella* Enteritidis, in addition to cross-protective responses against *S.* Typhimurium (16). These and other successful immunization efforts have led us to develop an EV-based approach against NTS that can be potentially translated and optimized for further human applications.

The antimicrobial activity of EVs derived from host immune cells has been exhibited both *in vitro* and *in vivo* (17, 18). One of the main reasons for this is the ability of these EVs to encapsulate and carry pathogen-derived antigens. (19). The inflammatory impact of intraluminal EV cargo can be context dependent and remains to be further characterized. The pro-inflammatory capacity of sEVs carrying pathogen-associated antigenic cargo has been demonstrated in both *Salmonella* Typhimurium and *Mycobacterium tuberculosis* infection models (11, 20). In previous studies, mice receiving sEVs from *Salmonella-*infected macrophages intranasally have demonstrated prolonged survival upon *Salmonella* challenge relative to the control group. Additionally, the bacterial burden in the liver of mice four days post *Salmonella* challenge was reduced significantly in mice receiving sEVs, compared to those not receiving intranasal sEVs (12). Additionally, intranasal sEV immunization also enhanced systemic IgG and mucosal IgA responses against the outer membrane proteins OmpA and OmpD—*Salmonella*-encoded antigens identified within sEVs isolated from RAW264.7 macrophages at 24- and 48- hours post-infection via mass spectrometry (11, 12). *Salmonella*-specific IgAs and IgGs produced in such vaccinated animals were also able to cross-react against heterologous *Salmonella* species derived from environmental sources (21). In parallel, several studies have demonstrated the beneficial effect of outer membrane vesicles (OMVs) as potential acellular vaccines for various applications (22, 23). Moreover, OMVs from flagellin-deficient *S.* Typhimurium administered through either the intranasal or intraperitoneal route elicited strong antibody responses and provided cross-protection against other *Salmonella* strains such as *S.* Enteritidis and *S.* Choleraesuis (24). These findings reinforce the broader concept that EV-based vaccines—whether host-derived sEVs or pathogen-derived OMVs—can stimulate protective immunity through either complementary or independent immunization mechanisms.

Our findings, along with emerging data that EVs can survive enzymatic degradation and acidic pH in the gastrointestinal tract, further support their feasibility as orally delivered vaccines (25). Bacterial-derived OMVs from *Vibrio cholerae*, *Helicobacter pylori*, and *Acinetobacter baumannii* when delivered orally induced protective immune responses *in vivo* [as reviewed in (26)]. Orally delivered antibiotic loaded OMVs from *A. baumannii* were able to exert bactericidal effects in the intestine of mice within 2 days post-delivery (27). More importantly, mesenchymal stem cell-derived EVs when targeted to the colon via oral administration, effectively alleviated ulcerative colitis *in vivo* (28). Although limited data exist on the *in vivo* fate of sEVs derived from mammalian immune cells following oral administration, we hypothesize that after surviving passage through the stomach, these *Salmonella-*antigen encapsulated sEVs may interact with microfold (M) cells in the Peyer’s patches in a manner similar to the bacterial antigens (29). This interaction could facilitate their transport via lymphatic or circulatory routes to distal organs, promote uptake by intestinal antigen-presenting cells (APCs) to initiate cell-mediated immune responses (28), or direct local B cells from the gut to migrate systemically to produce IgG antibodies (30). While we observed systemic IgG responses and improved survival, mucosal secretory IgA (SIgA) induction via oral administration was not tested in this study, due to its limited production in our previous observations (12). Prior work has reported that mice lacking the polymeric immunoglobulin receptor (pIgR) – important for secretory IgA (SIgA) transport to mucosal surfaces – had a reduced bacterial burden in tissues such as the liver and spleen following oral infection with *S.* Typhimurium. Furthermore, pIgR knockout mice showed improved survival following lethal *S.* Typhimurium infection, which indicates that mucosal IgA might not be necessary for long-term protective immunity against *Salmonella* (31). A progressive increase in serum IgG in immunized mice with specificity to *Salmonella* OmpA antigen, demonstrated to be present in sEVs from *Salmonella*-infected macrophages, indicated that orally delivered sEVs can boost production of antigen-specific protective antibody responses. Based on our presented results, orally administered sEVs elicited protective responses independently of robust SIgA generation. In line with previous experiments, our oral sEV immunization resulted in a decreased bacterial burden in the livers of vaccinated mice, and an overall improvement in these mice was also demonstrated by improved host resilience and reduced weight fluctuations. Elucidating the innate and adaptive immune mechanisms induced by sEVs administered through different mucosal routes (oral versus intranasal) is warranted to understand essential components that prime effective long-term immunological responses.

Future work will focus on optimizing oral sEV vaccine formulations for improved efficacy and cross-protective responses against heterologous strains. Strategies under consideration include encapsulation in enteric-coated carriers, co-delivery with mucosal adjuvants, and engineering of EV surface features to promote mucosal uptake and tropism for antigen presentation responses (32, 33). Since immune responses within the gut can vary significantly based on site-specificity within the tissues, it is important to account for these differences when designing nanoparticle-based therapies in order to develop precise, safe, and effective oral vaccines (34). This study was limited by the lack of assessment of cell-mediated immunity, which plays a key role in host defense against intracellular pathogens like *Salmonella* (11). Importantly, *Salmonella* pathogenesis involves inhibition of T cells, which impairs bridging responses necessary for long-term immunity (35). Additional mechanistic studies evaluating T cell-associated immune responses related to sEVs routes of administration, such as *ex vivo* stimulation of spleens and mesenteric lymph nodes (mLNs) of immunized mice to assess memory CD4 helper and CD8 cytotoxic T cell proliferation followed by intracellular cytokine detection, are required. A current limitation to this is that protective effects can only be explored in systemic organs, an issue that can be mitigated using a colitis-induced model for *Salmonella* infections. Overall, these results can further shed light into heterogenous protective memory-recall responses for optimization of immunizations strategies against salmonellosis.

In summary, this study demonstrates that orally administered sEVs from *S.* Typhimurium-infected macrophages confer significant protection against systemic *Salmonella* infection in mice. These findings further support the continued development of EV-based oral vaccines as a new and scalable approach for preventing bacterial enteric diseases.

## Data Availability

The original contributions presented in the study are included in the article/**Supplementary Material**. Further inquiries can be directed to the corresponding author.
