# Supplementary material for: Orally administered extracellular vesicles from Salmonella-infected macrophages confer protective immunity in vivo
Source: Front Immunol. 2025 Aug 15;16:1628756. doi: 10.3389/fimmu.2025.1628756 (PMC12394216; doi:10.3389/fimmu.2025.1628756)
Supplement: Supplementary file 1 [file DataSheet1.pdf]

Supplementary material

Supplementary

A

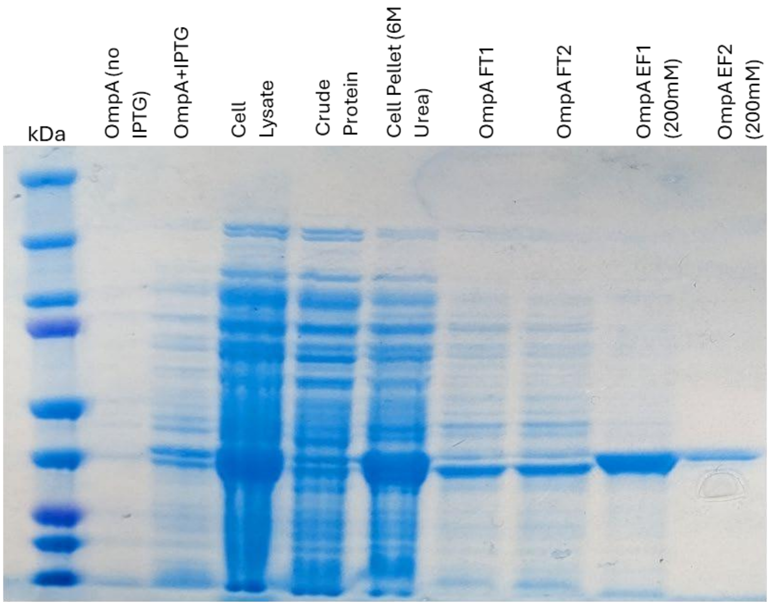

B

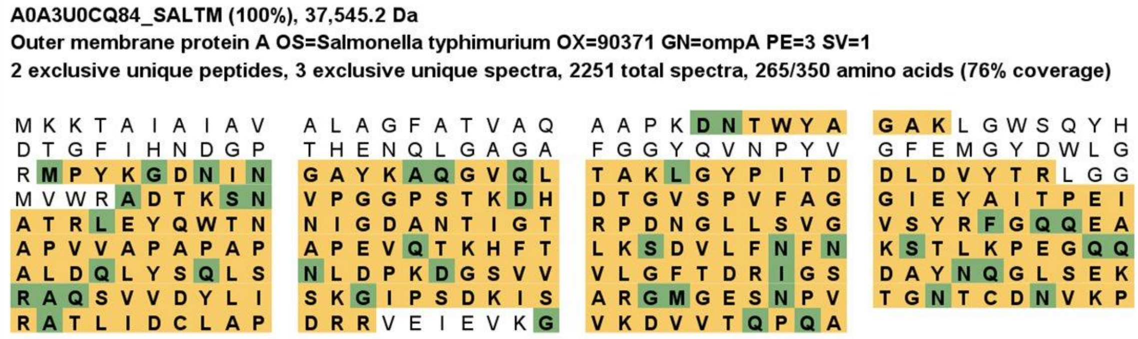

Figure S1. **(A)** SDS-PAGE showing purified recombinant OmpA from *S.* Typhimurium. OmpA eluted fraction (EF) 1 and 2 were collected using elution buffer containing 200 mM imidazole. **(B)** The band was excised, digested with trypsin as previously described *(1)*, followed by proteomic analysis of peptides using timsTOF flex (Bruker) mass spectrometer.

Supplementary References

1. Ocampo, J., Barker, H., Rice, K.C., and Ferraro, M.J. (2024). Impact of payload shielding on viability and proteomic profile: Insights from a stratospheric weather balloon flight experiment. *Gravitational and Space Research* 12, 64-76. doi:10.2478/gsr-2024-0005.
